# Supplementary material for: Saliva Diagnosis as a Disease Predictor
Source: J Clin Med. 2020 Jan 30;9(2):377. doi: 10.3390/jcm9020377 (PMC7074189; doi:10.3390/jcm9020377)
Supplement: Supplementary file 1 [file jcm-09-00377-s001.pdf]

Supplemental Tables

Supplemental Table 1. Salivary Abeta42 levels.

**Low Control**				
**Case No.**	**Age**	**Gender**	**Diagnosis**	Aβ (pg/ml)
1	45	F	N	22.56
2	54	M	N	22.32
3	40	F	N	19.63
4	15	M	N	18.66
5	53	F	N	20.56
6	89	F	N	19.73
7	88	M	N	22.15
8	55	M	N	20.93
9	32	F	N	23.04
10	42	F	N	20.57
11	45	M	N	22.48
12	45	F	N	22.46
13	54	M	N	19.93
14	10	M	N	21.54
15	56	M	N	22.81
16	76	M	N	22.21
17	88	F	N	19.83
18	62	F	N	20.34
19	83	F	N	20.56
20	18	M	N	22.49
21	57	M	N	21.94
22	53	F	N	22.58
23	40	F	N	20.82
24	17	M	N	21.47
25	46	M	N	21.45
26	54	M	N	19.58
27	81	M	N	21.37
28	57	F	N	23.07
29	81	M	N	19.54
30	59	F	N	20.23
31	24	M	N	19.34
32	54	F	N	20.59
33	56	M	N	21.66
34	78	F	N	22.28
35	42	F	N	20.45
36	34	F	N	20.48
37	35	M	N	19.83
38	54	M	N	24.44
39	48	M	N	21.32
40	90	M	N	22.35
41	71	M	N	22.19
42	22	M	N	19.23
43	19	M	N	18.12
44	25	F	N	19.58
45	57	M	N	20.63
46	59	F	N	21.47
47	28	F	N	21.08
48	45	M	N	21.24
49	49	M	N	22.51

50	55	M	N	21.56
51	81	F	N	21.47
52	81	M	N	20.44
53	86	M	N	21.94
54	69	M	N	20.84
55	88	M	N	22.46
56	43	F	N	21.35
57	54	M	N	22.56
58	52	M	N	20.33
59	53	M	N	21.28
60	55	F	N	32.31
61	54	M	N	21.42
62	49	F	N	20.67
63	40	M	N	23.56
64	48	F	N	21.65
65	51	M	N	22.27
66	47	F	N	21.29
67	66	F	N	20.34
68	77	M	N	27.34
69	47	F	N	22.37
70	52	M	N	21.35
71	74	F	N	20.59
72	78	M	N	22.38
73	12	M	N	21.16
74	16	M	N	21.47
75	19	F	N	19.73
76	22	M	N	22.27
77	24	M	N	21.53
78	39	M	N	22.22
79	42	M	N	20.58
80	47	F	N	20.15
81	55	F	N	29.66
82	59	M	N	21.55
83	60	M	N	22.02
84	62	M	N	21.37
85	78	M	N	21.44
86	92	F	N	20.89
87	67	M	N	24.63
88	52	F	N	23.78
89	93	M	N	20.55
90	95	F	N	20.38
91	84	M	N	19.46
92	46	F	N	20.27
93	48	M	N	18.93
94	79	F	N	19.44
95	54	F	N	20.58
96	87	M	N	19.66
97	41	M	N	19.21
98	84	M	N	19.45
99	16	M	N	18.66
100	52	F	N	19.66
101	52	F	N	20.93
102	52	F	N	18.16
103	84	F	N	19.07
104	52	F	N	19.05
105	52	F	N	20.59

106	68	M	N	20.23
107	51	F	N	21.33
108	40	F	N	21.33
109	47	F	N	21.11
110	53	M	N	21.53
111	63	F	N	20.88
112	61	F	N	19.93
113	44	M	N	20.18
114	61	M	N	21.35
115	59	F	N	21.76
116	71	M	N	20.63
117	56	M	N	22.05
118	52	F	N	20.57
119	22	M	N	19.23
120	66	F	N	22.27
121	93	F	N	21.07
122	63	M	N	21.11
123	31	F	N	21.36
124	81	M	N	20.13
125	77	M	N	22.42
126	60	F	N	20.25
127	70	F	N	21.21
128	67	F	N	22.19
129	70	M	N	21.07
130	45	F	N	22.17
131	76	M	N	23.18
132	63	F	N	20.66
133	33	M	N	22.11
134	42	M	N	20.25
135	42	F	N	21.05
136	14	M	N	20.25
137	56	F	N	21.42
138	76	F	N	22.16
139	43	F	N	20.94
140	58	M	N	21.18
141	63	M	N	22.17
142	51	F	N	21.14
143	75	F	N	22.06
144	74	M	N	20.36
145	76	M	N	19.62
146	79	F	N	21.26
147	47	F	N	20.94
148	40	F	N	22.43
Mean ± SD				21.26 ± 1.73
		High Control		
1	22	F	N	55.79
2	73	M	N	35.86
3	75	M	N	30.94
4	91	F	N	64.35
5	79	M	N	45.28
6	49	M	N	42.46
7	12	M	N	55.37
8	22	F	N	55.79
9	73	M	N	35.86
10	55	F	N	40.44
11	75	M	N	30.94

12	49	F	N	55.79
13	70	M	N	31.48
14	91	F	N	64.35
15	79	M	N	45.28
16	71	M	N	31.47
17	76	M	N	52.45
18	49	M	N	42.46
19	69	F	N	32.73
20	45	M	N	30.34
21	57	F	N	47.96
22	77	F	N	52.45
23	72	F	N	48.31
24	71	M	N	41.83
25	59	M	N	32.68
26	60	F	N	41.12
27	64	M	N	39.66
28	58	M	N	32.27
29	49	M	N	39.94
30	76	F	N	34.63
31	79	F	N	32.55
32	80	M	N	31.69
33	44	M	N	30.18
34	52	F	N	31.44
35	76	F	N	34.57
36	79	F	N	37.16
37	72	M	N	35.91
38	56	F	N	48.33
39	51	F	N	51.25
40	71	F	N	41.18
41	64	F	N	28.84
42	72	M	N	48.44
43	49	M	N	30.95
44	63	M	N	35.22
45	71	F	N	41.17
46	59	M	N	38.88
47	79	M	N	42.91
48	44	F	N	40.16
49	66	M	N	38.11
50	74	F	N	35.96
51	86	M	N	46.91
52	21	M	N	31.28
53	25	F	N	38.17
54	53	F	N	40.16
55	62	F	N	35.48
56	41	M	N	37.72
57	58	M	N	41.12
58	69	M	N	30.27
59	71	F	N	28.93
60	73	F	N	31.46
61	75	M	N	32.89
62	54	F	N	33.42
63	49	F	N	35.37
64	49	F	N	31.17
65	49	F	N	31.47
66	69	F	N	34.44
67	24	F	N	37.45

68	25	M	N	33.14
69	55	F	N	35.17
70	48	M	N	38.87
71	78	F	N	37.84
72	44	F	N	33.16
73	49	M	N	35.86
74	47	F	N	31.27
75	65	M	N	32.47
76	41	F	N	38.97
77	54	F	N	29.22
78	47	F	N	37.83
79	52	M	N	33.14
80	58	F	N	29.59
81	76	F	N	31.12
82	59	F	N	30.78
83	44	M	N	33.28
84	14	F	N	29.11
85	53	F	N	27.73
86	65	M	N	35.73
87	92	F	N	41.19
88	55	F	N	29.55
89	59	M	N	30.93
Mean ± SD				37.96 ± 8.13
AD				
1	60	F	AD	47.96
2	86	F	AD	75.2
3	57	M	AD	41.58
4	52	F	AD	59.57
5	77	F	AD	84.97
6	84	F	AD	64.93
7	86	F	AD	69.83
8	78	M	AD	44.22
9	72	F	AD	42.33
10	91	M	AD	52.17
11	84	M	AD	49.94
12	78	F	AD	55.93
13	75	F	AD	47.93
14	75	F	AD	60.68
15	74	M	AD	63.73
16	8	M	AD	42.77
17	72	F	AD	45.95
18	64	F	AD	53.38
19	48	F	AD	49.26
20	61	M	AD	62.44
21	78	F	AD	58.28
22	83	M	AD	44.19
23	80	F	AD	51.11
24	79	M	AD	49.07
25	84	F	AD	46.62
26	74	F	AD	44.62
27	80	M	AD	55.83
28	74	F	AD	59.66
29	81	F	AD	41.62
30	78	M	AD	55.18
Mean ± SD				51.70 ± 10.50

Supplemental Table 1. Abeta42 levels of low level control group (less than 23 pg/ml), high level control group (higher than 30 pg/ml) and AD were tested. N: normal group and AD: Alzheimer disease group. Averages and SD were 21.26 ± 1.73 for low level controls (148 cases), 37.96 ± 8.13 for high level controls (89 cases) and 51.70 ± 10.50 for AD cases (30 cases). One-way ANOVA was carried out to test significance for data. Multiple group comparisons were followed by a *post-hoc* Bonferroni t-test.; $P<0.01$ between low level control group and, high level control group and AD group and $P<0.01$ between high level control groups and AD group.

Supplemental Table 2. Salivary CRP levels.

Case No.	Age	Gender	Diagnosis	CRP (ng/ml)
		Low level control group		
1	35	M	N	1.97
2	28	F	N	1.94
3	86	M	N	1.88
4	66	F	N	1.88
5	47	F	N	1.88
6	78	F	N	1.85
7	57	M	Type II diabetes	1.83
8	81	F	Parkinson's Disease	1.84
9	52	M	N	1.83
10	42	F	N	1.81
11	87	M	N	1.79
12	59	F	N	1.77
13	22	F	N	1.74
14	49	F	N	1.74
15	12	F	N	1.74
16	53	M	N	1.74
17	24	M	N	1.73
18	55	M	N	1.72
19	55	F	N	1.67
20	56	M	N	1.59
21	74	F	N	1.59
22	73	M	N	1.58
23	48	F	N	1.58
24	47	F	N	1.58
25	59	F	N	1.47
26	43	F	N	1.47
27	54	M	N	1.44
28	90	M	N	1.42
29	54	M	N	1.32
30	48	M	N	1.29
31	89	F	N	1.12
Ave and SEM				1.67 ± 0.21
		High level control group		
1	79	M	N	8.82
2	12	M	N	8.37
3	88	M	N	7.92
4	70	M	N	7.45
5	71	M	N	7.37
6	83	M	N	6.26
7	55	F	N	5.93
8	52	M	N	5.93
9	34	F	N	5.85
10	73	F	N	5.84
11	49	F	N	5.83
12	54	M	N	5.72
13	40	M	N	5.72
14	77	M	N	5.58
15	78	M	N	5.55
16	49	M	N	4.84
17	45	M	N	4.57
18	35	F	N	4.45
19	53	M	N	3.62
20	45	M	N	3.58

21	75	M	N	3.48
22	71	M	N	3.48
23	71	F	N	3.37
24	51	M	N	3.17
25	49	M	N	3.16
26	59	M	N	3.11
Ave and SEM				5.34 ± 1.70
		Diseased Group		
1	84	M	Heart Attack	32.70
2	88	M	TIA	16.95
3	59	F	Peripheral nerve failure	16.45
4	69	M	Heart Attack	15.47
5	69	F	Heart Attack	14.66
6	81	M	Heart Attack	11.48
7	76	M	Heart attack	9.93
8	77	F	Giant cell Arthritis	9.93
9	54	M	Heart Attack	9.21
Ave and SEM				15.20 ± 7.21

Supplemental Table 2. C-reactive proteins (CRPs) levels of low controls, high controls and diseased conditions were tested. Averages and SD were 1.67 ± 0.21 for low level control group (31 cases), 5.34 ± 1.70 for high level control group (26 cases) and 15.20 ± 7.21 for diseased conditions (9 cases). One-way ANOVA was carried out to test significance for data. One-way ANOVA was carried out to test significance for data. Multiple group comparisons were followed by a *post-hoc* Bonferroni t-test; $P<0.01$ between low level control group and, high level control group and diseased group and $P<0.01$ between high level control group and diseased group.

Supplemental Table 3. Salivary TNF levels.

Case No	Age	Gender	Diagnosis	TNF (pg/ml)
		Control		
1	54	F	N	155.93
2	52	M	N	168.11
3	53	M	N	161.49
4	55	M	N	166.48
5	43	F	N	174.98
6	79	M	N	162.69
7	54	M	N	164.12
8	49	F	N	156.17
9	71	M	N	152.79
10	59	M	N	157.92
11	77	M	N	165.34
12	45	M	N	156.34
13	47	F	N	143.35
14	52	M	N	166.51
15	74	F	N	156.29
16	78	M	N	146.93
17	12	M	N	144.48
19	63	F	N	159.62
20	59	M	N	161.44
21	71	M	N	162.56
22	59	M	N	155.48
23	52	F	N	161.61
24	58	M	N	158.37
25	22	M	N	161.26
Ave and SEM				159.18 ± 7.38
		Cancer cases		
1	76	M	Leukemia	474.83
2	63	M	Lung cancer	296.44
3	70	M	Prostate cancer	311.36
4	81	M	Liver cancer	286.47
5	88	M	Colon Cancer	411.17
6	57	F	Ovarian cancer	397.17
7	65	M	Pancreatic Cancer	405.56
8	89	F	Glioma	431.66
9	54	F	Breast Cancer	295.63
10	67	M	Colon CSancer	406.68
11	70	M	Pancreatic cancer	288.47
12	70	M	Pancreatic Cancer	326.45
13	71	M	Prostate Cancer	368.37
14	64	M	Prostate Cancer	362.46
15	83	F	Breast Cancer	260.36
16	76	M	Stomach Cancer	378
17	77	M	Multiple Myeloma	275
Ave and SEM				351.53 ± 63.8

Supplemental Table 3. TNF levels of control group and cancer cases were tested. Averages and SEM were 159.18 ± 7.38 for control group (25 cases) and 351.53 ± 63.8 for cancer cases (17 cases). The table shows salivary TNF levels for all the cancer cases suffering from advanced cancer. The values ranged from 260 pg/ml to 474 pg/ml. There was no overlap between normal and cancer cases. One-way ANOVA was carried out to test significance; $P<0.01$ between control group and cancer cases.
